# Implementation challenges and perception of care providers on Electronic Medical Records at St. Paul’s and Ayder Hospitals, Ethiopia

**DOI:** 10.1186/s12911-021-01670-z

**Published:** 2021-11-02

**Authors:** Alemayehu Bisrat, Dagne Minda, Bekalu Assamnew, Biruk Abebe, Teshome Abegaz

**Affiliations:** 1 Library and Info Service Directorate, St. Paul’s Hospital Millennium Medical College, PO Box 1271, Addis Ababa, Ethiopia; 2 ICT Directorate, St. Paul’s Hospital Millennium Medical College, Addis Ababa, Ethiopia; 3 Medical Education Unit, St. Paul’s Hospital Millennium Medical College, Addis Ababa, Ethiopia; 4 Health Informatics and Healthcare Innovation Department, School of Public Health, College of Health Sciences, Mekele University, Mekele, Ethiopia

**Keywords:** Electronic Medical Record, Medical record, EMR implementation, Ethiopia

## Abstract

**Background:**

In resources constrained settings, effectively implemented Electronic Medical Record systems have numerous benefits over paper-based record keeping. This system was implemented in the 2009 Gregorian Calendar in the two Ethiopian territory hospitals, Ayder and St. Paul’s. The pilot implementation and similar re-deployment efforts done in 2014 and 2017 Gregorian Calendar failed at St. Paul's. This study aimed to assess the current status, identify challenges, success factors and perception of health care providers to the system to inform on future roll-outs and scale-up plans.

**Methods:**

A cross sectional study design with quantitative and qualitative methods was employed. A survey was administered October to December 2019 using a structured questionnaire. A total of 240 health care providers participated in the study based on a stratified random sampling technique. An interview was conducted with a total of 10 persons that include IT experts and higher managements of the hospital. Descriptive statistics were employed to summarize the survey data using SPSS V.21. Qualitative data were thematically presented.

**Results:**

St. Paul’s hospital predominantly practiced the manual medical recording system. The majority of respondents (30.6%) declared that a lack of training and follow up, lack of management commitment, poor network infrastructure and hardware/software-related issues were challenges and contributed to EMR system failure at St. Paul’s. Results from the qualitative data attested to the above results. The system is found well-functioning at Ayder, and the majority of respondents (38%) noted that lack of training and follow-up was the most piercing challenge. As per the qualitative findings, ICT infrastructure, availability of equipment, incentive mechanisms, and management commitment are mentioned as supportive for successful implementation. At both hospitals, 70 to 95% of participants hold favorable perceptions and are willing to use the system.

**Conclusion:**

Assessing the readiness of the hospital, selecting and acquiring standard and certified EMR systems, provision of adequate logistic requirements including equipment and supplies, and upgrading the hospital ICT infrastructure will allow sustainable deployment of an EMR system.

**Supplementary Information:**

The online version contains supplementary material available at 10.1186/s12911-021-01670-z.

## Background

Information technology has transformed nearly every single side of our lives. Over the past two decades, particularly in the healthcare system, it has been seen as a key and first step for improving the quality of health care service and patient safety [1]. Health information technology (HIT) has defined as “the application of information processing involving both computer hardware and software that deals with the storage, retrieval, sharing, and use of health care information, data, and knowledge for communication and decision making” [2]. These technologies have been promoted as potential tools for improving quality, reducing cost, and improving the efficiency of the health care system [2]. Studies revealed that HIT offered several opportunities for improving and changing health care. These include; reducing human errors, improving medication safety, improving clinical outcomes, facilitating care coordination, improving practice efficiencies, increasing patient satisfaction, and tracking data over time [1, 3].

Among the commonly used health information technology tools intended to improve practice efficiency and patient care, one is Electronic Medical Record. WHO [4] described EMR as a digital version of all the information typically found in a provider’s paper chart: medical history, diagnoses, medications, immunization dates, allergies, lab results and doctor’s notes [4]. Other studies described EMR as an individual health related information within one health care organization and an essential component of a robust and efficient modern healthcare system [5, 6].

Studies identified several reasons for the need of adopting EMR in hospital settings. EMR systems would help achieve improved patient care in terms of safety, efficiency, and quality [1]. It was noted that adopting and implementing EMR can allow easy access to patient information, reduce error, improved quality of care, improved documentation, save time, and improve order and receipt of lab tests as well as diagnostic images [5, 7, 8]. On the other hand, EMR systems facilitate the management of appointments, scheduling, registration, admission, discharge, and transfer of patients [9].

Paper-based hospital record-keeping and workflows dependent on paper have proven to become more and more inefficient and are continuously failing to meet the care providers and patient needs. Paper records were criticized for their limited accessibility and their general incompleteness [8, 10]. Paper-based systems are considered a human resource and time-intensive and are often plagued by inaccurate reporting processes leading to out-of-date and irrelevant data [11]. Other studies were concluding that patient data in paper records often are vague, ambiguous, incomplete, or hard to extract information from its illegible handwritten pages, has limited opportunities for reuse and sharing of data [10–12].

In spite of some implementation challenges that lead to the failure of pilot projects, EMR systems have been shown to be feasible and documenting numerous benefits, such as medical error prevention, reduction of unnecessary care costs, and hold the greatest promise for improving the quality of health care services. Several studies noted, EMR systems enhance the quality of care, improve communication, decrease patient waiting time, are able to check for drug allergies, calculate doses and help to reduce medical errors [6, 10, 13, 14].

Other studies identified barriers hampering the implementation of EMR in low and middle-income countries. These include limited funding, lack of full-time IT experts, poor information technology infrastructure, lack of automatic data and power backups, and a need to modify the existing workflow processes [3, 15, 16]. Similarly, an EMR deployment requires the availability of appropriate information systems infrastructures, such as reliable power, connectivity, and networking capabilities [9]. Human barriers, including negative beliefs, behaviors, and attitudes of healthcare professionals towards such systems, were mentioned as the most prevalent barriers [17]. Lack of technical expertise, funding, an integrated EMR system, and physicians' time also has been identified as barriers obstructing EMR implementation [11, 18].

Despite the concerns and interest in adopting and implementing Electronic Medical Records in our country, there is a large gap between planning to use this system in hospitals and operating them ideally to achieve its purpose and expected benefits. The Federal Ministry of Health has begun the adoption of SmartCare since the 2009 Gregorian Calendar. SmartCare was later renamed Tena Care and became a comprehensive EMR system. The US Centers for Disease Control and Prevention (CDC) initially, developed the system and later on customized and maintained by IT staff and medical doctors of the Tulane University Technical Assistance Project in Ethiopia (TUTAPE). The deployment took place at Ayder and St. Paul’s hospitals in the 2009 Gregorian Calendar. While following unsuccessful implementation efforts, consecutive redeployment took place at St. Paul’s in 2014 and 2017 Gregorian Calendar. The overall objectives of implementing an EMR in these hospitals were to make health information accessible at the point of service and improve information use, avoid duplicate medical records for a single patient, minimize the shelves used by paper medical records, increase the quality of care delivery and finally save hospital costs. Unfortunately, pieces of evidence on EMR implementation challenges, factors contributing to the success or failure were not found, and evaluation of the systems capability and quality is also meager.

This study aimed to assess the status of the EMR system, identify challenges, facilitators and barriers, while at the same time examine the perception of health care providers toward the system comparing Ayder and St. Paul’s teaching hospitals to draw a lesson for sustainable EMR deployment at local settings.

The study addressed questions related to what significant contributions did the system brought in health service delivery concerning the two hospitals' performance? What are the success factors, challenges, or impediments, and how do health care providers perceived EMR implementation?

## Methods

### Location and settings of the study

The study was carried out at the biggest referral hospitals in the country where a high volume of patients attends emergency and outpatient clinics daily for advanced medical care and treatment. These are St. Paul’s hospital, Addis Ababa, and Ayder hospital, Mekele, where an EMR system was implemented, with the support of the FMoH and the regional health bureau.

St. Paul’s hospital was established in 1968 Gregorian Calendar by the late Emperor Haile Selassie and re-organized as St Paul’s Hospital Millennium Medical College through a decree of the Council of Ministers in 2010 Gregorian Calendar, though the medical school opened in 2007 Gregorian calendar. It is governed by a board, under the Federal Ministry of Health.

It has more than 2800 clinical, academic and administrative support staff that provide medical specialty services to patients referred from all over the country, teaching medicine and nursing students and doing basic and applied researches. While the inpatient capacity is more than 700 beds, the hospital sees an average of 1200 emergency and outpatient clients daily.

Ayder Comprehensive Specialized Hospital commenced rendering its referral and non-referral services in 2008 Gregorian Calendar to the 9 million population in its catchment areas of the Tigray, Afar, and North-eastern parts of the Amhara Regional States including the Eritrean refugees.

The hospital can be designated as the most advanced medical facility by all accounts in the Northern part of the country, and it stands as the second-largest hospital in the nation with a total capacity of about 500 inpatient beds in all departments and other specialty units. It is used as a teaching hospital and research center for the College of Health Sciences, Mekelle University. It has above 80 specialists, in various areas of medical specializations and fairly adequate numbers of all the other health professionals constituting the health care team.

### Survey design

The study design used both quantitative and qualitative approaches. The survey employed a structured and self-completion questionnaire. It used a stratified random sampling technique. For the qualitative part of the study, a purposive sampling technique was applied to interview key informants such as IT staff, EMR system administrators, medical directors, and hospital provosts.

### Data sources

The study was limited to respondents serving in St. Paul’s Hospital Millennium Medical College and Ayder referral hospital. The study population covers Specialists, General Practitioners, Nurses, Laboratory Scientists and Pharmacists working full time in both St. Paul’s i.e. 1400, and Ayder hospitals i.e. 1000 that totals 2400. Besides, an in-depth interview with key informants that include 3 IT staff working in relation to EMR systems and 2 staff from the senior managements from each hospital was conducted.

### Sample size

The sample size of 100 and 140 from Ayder and St. Paul’s respectively representing approximately 10% of the study population was selected using a table of random figures. The total sample size was 240.

The formula for calculating the sample size was:

$${\text{nr}} = \frac{{4{\text{pq}}}}{{{\text{d}}^{2} }}$$

where nr = required sample size, p = proportion of the population having the characteristic, q = 1-p and d = the degree of precision (for this case a 10% margin of error i.e. d = 0.1).

### Data collection procedure

A structured and pre-validated questionnaire was used which contained sections on Socio-Demographics; Knowledge, Perceived Benefits and Usefulness; and Perception and Willingness (Additional file 1).

The perception was assessed using 5 perception items measured by a five-point Likert scale ‘Strongly Disagree = 1’, ‘Disagree = 2’, Don’t Know = 3’, ‘Agree = 4’ and ‘Strongly Agree = 5’. Higher scores i.e. 4 and 5 represented a more positive perception towards EMR and willingness to use the system.

The questionnaire was pre-tested before the actual study, on a hospital outside of the study area, and adjusted and proved for its consistency and reliability. The survey was administered October through December 2019 with the help of 2 data collectors and supervised by the investigators. To support the data obtained from the questionnaire with a more detailed story on the system an interview was conducted with key informants including the IT staff, EMR system administrators, higher hospital management with the help of a pre-distributed interview guide (Additional file 1). To ensure the trustworthiness of the findings, we tried to use triangulation that showed its credibility. We also tried to control the researcher's bias or personal motivation not to alter the interpretation. The research process and data analysis were reviewed and examined to ensure the findings are consistent and could be repeated. Descriptive statistics and chi-square tests were employed to summarize the data and explored associations between variables using SPSS V.21. Results from the qualitative data were categorized and presented.

## Results

A total of 172 participants (100 from Ayder and 72 from St. Paul’s), out of 240 health care providers selected for the study, were responded to the survey that made a response rate of 71.7%. Among the study participants, 56.4% were female, and 43.6% were male (Table 1). It was found that 53.5% of participants were in the age group of 21–30 years, while 34.9% were in the age group of 31–40 years old. Sixty-Seven (39%) of them have served for 6–10 years, and sixty-five (37.8%) have 1–5 years of service. Among the participants, 6.4% were General Practitioners, 18% were Specialist, 61.6% were Nurses, 5.8% were Lab Technicians or laboratory scientists, and 8.1% were Pharmacists. The majority of the respondents were nurses, which were 50% at St. Paul’s and 70% at Ayder, followed by specialists, which were 29.2% at St. Paul’s and 10% at Ayder.

**Table 1:** Socio-demographic characterstics of respondents by institution

Item	Respondents Institution			
	St. Paul's Hospital		Ayder Hospital	
	Freq	%	Freq	%
Sex				
Male	40	55.6%	35	35.0%
Female	32	44.4%	65	65.0%
Age range				
21–30	35	48.6%	57	57.0%
31–40	25	34.7%	35	35.0%
41–50	6	8.3%	7	7.0%
51–60	6	8.3%	1	1.0%
Professional category				
GP	8	11.1%	3	3.0%
Specialist	21	29.2%	10	10.0%
Nurse	36	50.0%	70	70.0%
Lab technician	3	4.2%	7	7.0%
Pharmacist	4	5.6%	10	10.0%
Service year				
1–5	33	45.8%	32	32.0%
6–10	23	31.9%	44	44.0%
11–15	10	13.9%	16	16.0%
> 15	6	8.3%	8	8.0%

**Fig. 1:**
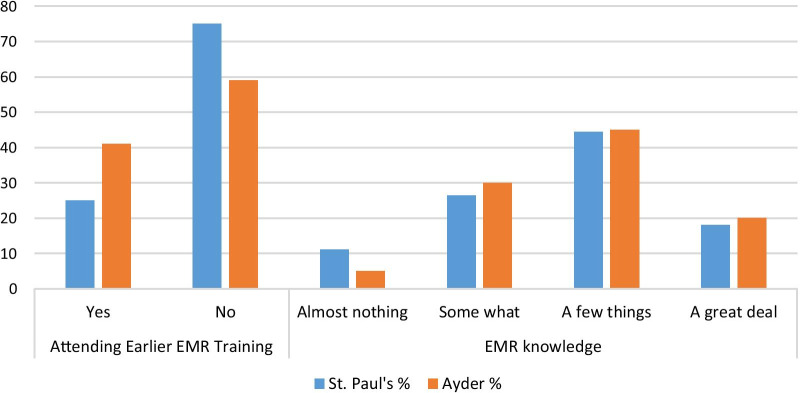
Participants past EMR training and system knowhow

For a question assessing their earlier training attendance on the system, 75% of St. Paul’s and 59% of Ayder respondents were not attended past EMR system training (Fig. 1). Regarding their know-how of the EMR system, 44.4% of St. Paul’s and 45% of Ayder respondents knew a few things about the system (Fig. 1).

A chi-square test was employed to test the existence of a relationship between professional categories vis-a-vis respondents' knowhow of the EMR system (Table 2), mean-while, the tests indicated that there was no significant association between the variables.

**Table 2:** Chi-square test of respondent’s professional category and know-how of the EMR system

Test	Value	*df*	Asymp. Sig. (2-sided)
Pearson Chi-square	14.668^a^	12	.260
Likelihood ratio	18.458	12	.102
Linear-by-linear association	.581	1	.446
N of valid cases	172		

Findings showed the majority of respondents from St. Paul’s viewed using the EMR system as a potential to change the process of patient care significantly (84.7%), and for 62% of respondents from Ayder the EMR already significantly changed the process of patient care (Table 3). The system has the potential to improve the quality of care for 93.1% of St. Paul’s respondents, and 84% of Ayder respondents reported that EMR already improved the quality of care. Further, sixty (83.3%) of St. Paul’s and Eighty-five (85%) of Ayder respondents agreed it has a benefit of increasing practice productivity (patient per day). For 83.3% of St. Paul’s and 78% of Ayder respondents' EMR has the benefit of decreasing workload and enhancing the efficiency of care providers. As per the finding, sixty-three (87.5%) of St. Paul’s and eighty-six (86%) of Ayder participants responded that patient records available on EMR systems are safer and more secure than paper-based medical recording systems.

**Table 3:** Views of respondents on the benefit of the EMR system

Items	Respondents Hospital			
	St. Paul's		Ayder	
	Freq	%	Freq	%
EMR will change /already changed the process of patient care?				
Significantly	61	84.7%	62	62.0%
Small degree	7	9.7%	25	25.0%
Not at all	4	5.6%	13	13.0%
EMR will change/already changed the quality of care?				
Will Improve	67	93.1%	84	84.0%
Decrease	0	0.0%	9	9.0%
No change	5	6.9%	7	7.0%
EMR has a benefit of increasing practice productivity				
Yes	60	83.3%	85	85.0%
No	12	16.7%	15	15.0%
EMR has a benefit of decreasing the work load and enhancing efficiency of providers				
Yes	60	83.3%	78	78.0%
No	12	16.7%	22	22.0%
EMR system is safe and secured as compared to a paper based medical record system				
Yes	63	87.5%	86	86.0%
No	9	12.5%	14	14.0%

**Fig. 2:**
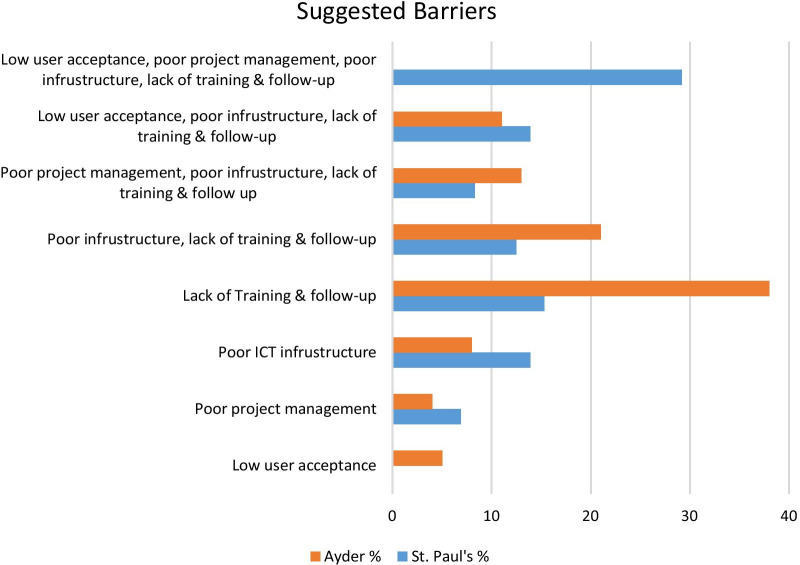
Suggested barriers to Implementation of EMR systems

**Fig. 3:**
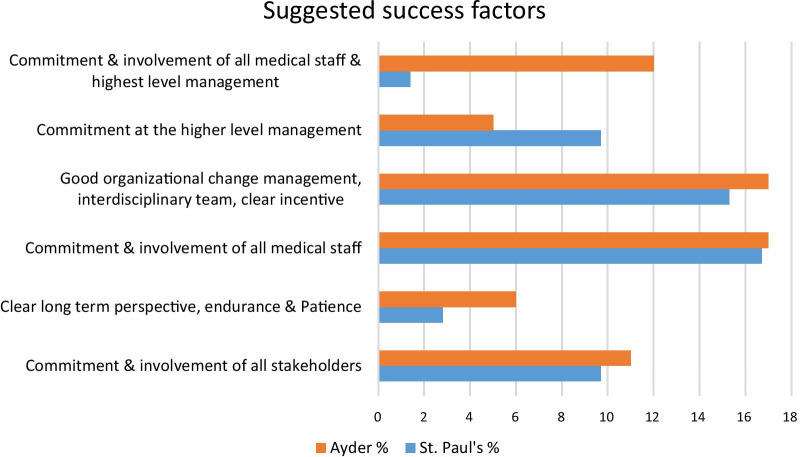
Suggested factors for successful implementation of EMR systems

Respondents identified barriers and success factors to the implementation of an EMR system. For 29.2% of St. Paul’s respondents suggested barriers were low user acceptance, poor project management, poor ICT infrastructure, and lack of training and follow-up while for 38% of Ayder respondents lack of training and follow-up were major challenges to the system implementation (Fig. 2). Furthermore, 21% of Ayder respondents suggested that poor ICT infrastructure and lack of training and follow-up were barriers to system implementation, while 15.3% of St. Paul’s respondents suggested lack of training and follow-up as barriers. On the other hand, 44.4% of Ayder and 32% of St. Paul’s respondents suggested commitment and involvement of all medical staff together with good organizational change management, an interdisciplinary team having IT experience, training and incentive mechanisms would result in successful implementation (Fig. 3).

Based on their actual day-to-day clinical practice 88% of Ayder respondents believed service turnaround time improved or decreased by using the EMR system, and 75% of the respondents assumed the improvement had ranging from thirty minutes to an hour (Table 4). Assessing EMR system downtime effect on clinical practices, 80% of Ayder respondents agreed it had a high to serious effect on their clinical practice. Regarding system effect on patients waiting time, 67% of Ayder respondents noticed a reduction in patient waiting time.

**Table 4:** Views on EMR system contributions to Service improvement at Ayder Hospital

Items	Respondents Ins	
	Ayder Hospital	
	Freq	%
Service turnaround time improved/ decreased by using EMR system		
Yes	88	88.0%
No	12	12.0%
By how much the time improved for the patient?		
By 30 min	39	39.0%
By 1 h	36	36.0%
By 2 h	8	8.0%
By More than 2 h	7	7.0%
Not applicable	10	10.0%
System down time affects clinical practice?		
Very minimum effect	12	12.0%
High effect	45	45.0%
Serious effect	35	35.0%
No effect at all	8	8.0%
EMR system effect on patients waiting time?		
Noted reduction of patient waiting time	67	67.0%
Increased patient waiting time	25	25.0%
No change	8	8.0%

In general, the assessment of the perception of participating health care providers showed positive insights. In both hospitals, findings of the study on perception towards the system indicated favorable perception, particularly on the easiness of the system, their preference to use it, completeness of patient information available on EMR, care providers interest for involvement at the design and implementation phase, and overall interest to use the system in future practice (Table 5).

**Table 5:** Respondents perception and willingness to use EMR for future clinical practice

Items	Respondents Institution			
	St. Paul's		Ayder Hospital	
	Freq	%	Freq	%
It would be easy for me to become skillful at using EMRs				
Strongly disagree	0	0.0%	5	5.0%
Disagree	5	6.9%	10	10.0%
Don't know	6	8.3%	7	7.0%
Agree	40	55.6%	55	55.0%
Strongly agree	21	29.2%	23	23.0%
I prefer EMR over paper charting				
Strongly disagree	1	1.4%	9	9.0%
Disagree	7	9.7%	9	9.0%
Don't know	3	4.2%	12	12.0%
Agree	32	44.4%	39	39.0%
Strongly agree	29	40.3%	31	31.0%
Patient information obtained from EMR is more complete as compared to Paper medical record				
Strongly disagree	0	0.0%	6	6.0%
Disagree	8	11.1%	23	23.0%
Don't know	11	15.3%	11	11.0%
Agree	28	38.9%	39	39.0%
Strongly agree	25	34.7%	21	21.0%
My involvement at the system design and during the EMR implementation phase will make the EMR more useful to me				
Strongly disagree	0	0.0%	4	4.0%
Disagree	1	1.4%	4	4.0%
Don't know	5	6.9%	9	9.0%
Agree	42	58.3%	65	65.0%
Strongly agree	24	33.3%	18	18.0%
I would like to use EMRs in my work in future				
Strongly disagree	0	0.0%	2	2.0%
Disagree	1	1.4%	4	4.0%
Don't know	2	2.8%	4	4.0%
Agree	26	36.1%	44	44.4%
Strongly agree	43	59.7%	45	45.5%

### Results from the qualitative data

The findings from the qualitative data explored overall views and detailed stories from the hospitals' management, EMR system administrators, and IT support personnel. These results were believed to reinforced the quantitative results and thus presented below. Further, a summary of general themes, sub-themes, and categories were presented (Table 6).

**Table 6:** Summary of qualitative data general themes, sub-themes and response categories

Themes	Sub-themes	Response categories
EMR implementation	Deployment objective	Improve service quality, efficiency, decrease hospital cost
	Current system status	Functioning at Ayder and failed at SPHMMC
		Dependency on the system at Ayder
		Using the manual system at SPHMMC
ICT infrastructure	IT facilities and equipment	Network infrastructure
		Availability of equipment and facilities
IT support theme	IT personnel	System administrators
		IT experts
Organization	Management commitment	Priority in the plan
		Insure readiness of the hospitals
		Participation of care providers
		Availability of champions
		Top level management commitment
		Training and follow up
Barriers/impediments	Human	Low staff awareness
		Resistance from the health workers
		Lack of skill
		Lack of training
		Work load on physicians
	Finance	Non availability of fund
	Organizational	Low level of management commitment
		Poor project management
		Change management
	Technical	Lack of equipment
		Lack of standard/certified EMR system
		Poor ICT infrastructure, slow network connection
		Lack of technical expertise/IT
		Lack of system Interoperability and integration
		Lack of ease of use
		Data security/vulnerability
Success factors/facilitators	Human	Management commitment
		Skilled manpower
		Change management
		Good project management, long term perspective
		Team sprit
		Training and follow up, motivation
		IT experts/system developers
		Availability of Champion
	Finance	Availability of fund
		Incentives mechanism
	Technical	Infrastructure, equipment
		Network speed
		Hardware and standard software availability

### EMR system implementation objectives, deployment efforts and current status

The result showed the management of both hospitals were planned to replace paper-based record keeping with the electronic recording system by "implementing the SmartCare EMR system, which was determined to improve the quality and efficiency and above all to reduce hospital costs” (Higher Hospital Management 001, 002, 006). As commented by interviewees, “this target was accomplished at Ayder, and the system is still well functioning. Currently, all medical services are becoming dependent on the system. We established an EMR committee, and in consultation with the representative from each department, we were able to maintain uninterruptable 24 h IT support” (Higher Hospital Management 001, 002).

At St. Paul’s, the SmartCare EMR project was initiated in 2009, though the implementation was done in phases. As the respondent explained, “the initial phase started at the medical record room where the bulk of data encoding takes place. Then other clinical areas like the outpatient department followed. But unfortunately, the operation failed in the middle of 2010 Gregorian Calendar”. The interviewee also added, "unsuccessful redeployment efforts made during the 2014 and 2017 Gregorian Calendar but the hospital was forced to practice predominantly with the traditional manual medical recording system – paper-based record-keeping” (Higher Hospital Management 006, 007, Sys Admin 008).

### ICT infrastructures and availability of technical support staff

The result revealed ICT infrastructure at Ayder was in good standing. The interviewee replied that “during deployment of SmartCare EMR system, staffs from TUTAPE in collaboration with FMoH was responsible for providing all technical support. These were, installing and configuring the software, supplying equipment, taking system backup, training the IT staff and care providers, and follow-up of the project” (Sys Admin 003).

The respondent further explored that “later on, the establishment of an ICT infrastructure across the hospital and the university with IT facilities, equipment’s and well-equipped data center, fortunately, bucked the SmartCare EMR implementation and contributed to have a sustainable system to the current date at Ayder” (Sys Admin 003). Another major shortcoming reported by interviewees at both hospitals was associated with the wireless network connectivity; “The network predominantly is based on the wireless network connection which is prone to interference by other networks and sometimes obstructs the connection. It also lacks the required speed and has security problems” (Sys Admin 003, Sys Admin 008).

However, St. Paul’s interviewee replied that “the hospital was lacking a well-developed IT infrastructure, facilities, equipment, and IT staff were almost nonexistent to take over the pilot work from TUTAPE which later resulted in the disappointment of users and the general collapse of the project” (Sys Admin 008, IT Support 009 and 010).

### Management commitment and future plan

Regarding top management support and level of awareness of care providers, the result revealed that “top management were very committed, but at the beginning of the system deployment there was resistance to it from the physician’s side while this improved after training and actual usage on the system” (Higher Hospital Management 002, Sys Admin 003).

The interviewee at St. Paul’s revealed, “the management was committed to prior efforts, and there were recent initiations to implement a new EMR system” (Higher Hospital Management 006, Sys Admin 008). The interviewee further suggested that “hospital-wide readiness assessment, acquiring appropriate and certified system, improving ICT infrastructure, equipment and facilities need consideration” (Sys Admin 003, Sys Admin 008, IT Support 010).

### Barriers/impediments to implementation

Findings from the senior management and IT experts’ side at St. Paul’s showed the existence of challenges that seriously impede former EMR implementation efforts. Quoted response from the interviewee presented as follows: “The challenges include intermittent connection with the network, poor network infrastructure, low staff awareness, low level of commitment from the top management, lack of integration and interoperability with other systems such as laboratory and PACS, IT support was almost nonexistent, there was no team/committee to coordinate or run the project, the software system lacks user-friendliness and there is resistance from the physicians not to use the system” (Sys Admin 008, IT Support 009 and 010).

Interview participants from Ayder hospital consented to the easiness of the software system but, they said that “initially, it was not designed in the way to meet the needs of the hospitals and end-users where it intended to serve. The system lacked interoperability as well as had maintenance issues. Fortunately, the developer at Ayder was initiated and worked on upgrading the system, making significant changes and major improvements to meet the requirements of end-users and today insured best performance system with the highest level of security” (Higher Hospital Management 002, Sys Admin 003, IT Support 004 and 005). “Wireless network connectivity, financing related to availing new computers and shortage of IT staff to respond to system-related emergencies at all corners of the hospital suggested still as obstacles at Ayder hospital” (Sys Admin 003, IT support 004).

### Success factors

Interview participants from Ayder hospital identified several points for system success in their hospital. The response from the interviewees quoted as follows; “The sustainability of the EMR system depends mainly on the existence of ‘individual champion’ in this case, the commitment of the system administrator (developer) was crucial, and any system associated problems solved easily. Further, the ICT infrastructure and availability of other equipment and facilities were supportive of the successful implementation. The commitment of top management, concern, willingness, and involvement of medical staffs and availability of incentives for IT support staff contributed a lot” (Higher Hospital Management 001, Sys Admin 003, IT Support 005).

## Discussion

An EMR system recognized in supporting a high-quality, integrated health care information system, which is independent of the place and time of health care delivery through information communication technology [19], is perceived to improve efficiency and increase the effectiveness of health care delivery [6, 10, 15, 20].

The two tertiary government hospitals were considered pioneers in introducing the EMR system at clinical settings and were assumed to transform the health care service with an improved flow of health information. The primary goals were achieving efficiency and effectiveness in the healthcare delivery system. Keeping structured information, minimizing medical errors, improving legibility of medical records, and in the meantime reducing hospital costs associated with the purchase of X-ray films, printing history sheets, lab orders, and other administrative expenses while supporting interoperability across information systems were aimed.

Deployment of the SmartCare EMR system took place in the 2009 Gregorian Calendar at Ayder hospital with support from TUTAPE, and FMoH was still functioning well. The initial version was upgraded and improved by the system developer of the hospital, and currently, Ayder has a better and dependable system to work on. While at St. Paul’s though similar EMR systems implemented by TUTAPE at different times and technical support availed at the initial phase were comparable, all efforts were not successful and the researchers of this study examined the hospital predominantly working on the traditional paper-based patient recording system.

The literature identified training and follow-up as essential elements of EMR actualization and need consideration before any large-scale EMR implementation [14, 21, 22]. The finding from one study emphasized that an important pre-requisite for the implementation of an EMR system is to learn at least the basics of how to function on the software system [21]. Our study identified 75% of St. Paul’s respondents had not attended previous EMR system-related training provided at the institution. This indicated the project failed at attaining one of the basic requirements to be fulfilled from the end-users’ side.

Similar kinds of literature disclosed introduction of EMR in the health care delivery system had enhanced quality of care, improved communication, reduced error, increased safety, decreased care costs, and improved capability of conducting research [6, 15, 23].

In a study conducted at a referral hospital in Kigali, Rwanda, which assessed health care consumers' perception of an EMR named OpenClinic, 88.2% of participants perceived the quality of care had improved since the introduction of OpenClinic EMR [19]. Similarly, 80.6% of the respondents supposed that the OpenClinic EMR had significantly changed the process of patient care positively, for instance, reduction in waiting time, improving the precision of record, improving safety and security of medical records [19]. One study concluded that, following the implementation of EMR in Kenya, patients spent less time waiting to see a consultant, and total time per visit at the health facilities was relatively shortened in duration [22].

In our study, the majority of participants confirmed similar findings as to the above researches. Service turnaround time is believed to be improved for 83.3% of St. Paul’s and improved in real practice for 88% of Ayder hospital respondents. There is an expectation in reduction of patient waiting time for the majority of St. Paul’s respondents (80.6%), while 67% of Ayder respondents attested actual reduction of patient waiting time using SmartCare EMR system for the last ten years.

The findings from this study assured, at Ayder hospital, the patient care process had changed significantly after the installation of the system. Quality of care had improved at Ayder for 84% of the respondents, implementation of SmartCare EMR had increased practice productivity, decreased the workload, and enhanced the efficiency of care providers. Concerning the safety of patient records, for sixty-three (87.5%) of St. Paul’s and eighty-six (86%) of Ayder respondents, patient records available on the EMR system are safer and more secure when compared to the paper-based medical recording system.

Several studies demonstrated issues considered as obstacles to the implementation of an EMR system, were mainly associated with a shortage of human resources, hardware and software failure, user resistance, lack of computer literacy, lack of technical expertise, lack of awareness, lack of understanding stakeholders demand, and lack of appropriate ICT infrastructure with facilities and equipment [10, 12, 15–18]. Cost, privacy concerns, lack of uniform standards, resistance, lack of policy support, vendor support and maintenance, and lacking awareness, were mentioned as obstructing EMR system implementation [9, 18, 19]. Further, as the literature explored, EMR implementation projects were inhibited fundamentally, with issues related to technical support, financing, procuring sufficient logistics, health professionals' beliefs and attitudes, electronic data exchange standards, and interoperability of the system [22, 24, 25].

The finding from qualitative and quantitative data of this study confirmed low user acceptance, poor project leadership, poor network connectivity, low staff awareness, lack of training and follow-up, and low level of commitment from the top management were the barriers to the system implementation and accounted for the failure of deployment and redeployment efforts in 2009, 2014 and 2017 Gregorian Calendar. Similarly, unfriendly user interface, lack of integration and interoperability with another system, unsatisfactory IT support, unavailability of a robust team to play the leadership role, and other hardware/software related issues were considered as an obstruction. Appropriate supports which were required for implementation and meaningful use of the EMR system was non-existent at St. Paul’s hospitals, did not have funding, there were no appropriate incentives for IT staff as well as end-users.

On the other hand, as some studies revealed, for successful EMR implementation; skilled IT personnel, training, infrastructures, change management, reliable system, effective project management, motivation, faster network, reliable data handling methods, and uninterrupted power supply, have vital importance [10, 24, 26, 27]. Our study revealed, beyond a well-established ICT infrastructure with the state-of-the-art technology data center, the commitment of all stakeholders and top management, involvement of care providers at the system design, testing, and implementation phase, and clear long-term perspective would result in successful implementation.

Another study identified some difficulties which are associated with wireless network connection stating that: it is prone to interference by other networks and wireless-enabled devices, objects such as walls can obstruct connection, it lacks the required data transmission speed, it is less secure, and ultimately these all compromise performance and quality of the network connection [28]. Similarly, in our study, the findings from the qualitative data explored problems associated with the existing network infrastructure at both hospitals, which is predominantly based on a wireless network and reported as it is a source of discomfort for end users. The result also disclosed that the Ayder hospital administration has started to address wired network connectivity problems at all places of the hospital, to ensure the sustenance of the system.

As the findings from the qualitative as well as quantitative study attested, at St. Paul’s hospital, the scenario was still unattractive for hospital-wide technology application. The management seems confident and eager to adopting innovative health information technologies to support clinical activities like the EMR. But the findings showed flourishing poor organizational ICT infrastructure, shortage of IT staff, and unavailability of standard software meeting the health information technology requirement that was considered as impeding earlier EMR deployments, hopefully, obstruct any future initiation efforts.

In general, the findings from this study attested how health information technology, particularly EMR, is changing the landscape in which health care service is being delivered locally. We can draw a lesson from this experience at Ayder that confirms a single health information technology application saved hospital costs of around 75,000 USD per year, which was expended only for the purchase of X-ray film for producing radiographic images. The existence of favorable ground such as well-equipped ICT infrastructure, top management commitment, uninterrupted technical support, technology-seeking behavior among care providers is also valuable lessons for St. Paul’s or other similar health care organizations at similar system implementation projects.

### Limitation of the study

The findings of this study have to be seen in light of some limitations. We understand that the sample size of the qualitative part is small and limited. Though we applied the purposive sampling technique and ensured the trustworthiness of the findings, this may prevent them from being extrapolated. We suggested further research with a large sample.

## Conclusion and recommendation

Implementing and practicing an EMR system at any tier of the health care system has been shown to increase medical practices’ productivity and quality of care. In resource-limited countries like Ethiopia, considering EMR for clinical care is no more a luxury. It is just being a requirement for providing modern standard care for patients and their families.

This research has studied the adoption and implementation of EMR systems at two tertiary care hospitals in Ethiopia-St. Paul’s and Ayder. The implementation outcomes are significantly different with achieving the initial objectives and maintaining a sustainable system where successful progress was observable at Ayder hospital.

Thus, primarily, assessing institutional and health professionals’ readiness, selecting and acquiring the best appropriate and certified EMR system meeting well-identified requirements of the local health care system, needs thoughtful attention. Adopting a sustainable EMR system at the hospital level further entails providing adequate logistics requirements such as equipment and facilities, reliable network connection, training and follow-up, power, and data backups. Besides, an IT and health care team, stakeholders’ engagement, top management commitment, and establishing ways and means for rewarding staff is overriding.

## Supplementary Information

**Additional file 1 Title of data**: Questionnaire and Interview guide for implementation challenges and perception of care providers on EMR at St. Paul’s and Ayder Hospitals, Ethiopia. **Description of Data**: A structured and pre-validated questionnaire which contains sections on Socio-Demographics; Knowledge, Perceived Benefits and Usefulness; and Perception and Willingness; whereas a pre-distributed interview guide employed to interview the key informants assuming to get more detailed information in support of findings from the questionnaire

## Data Availability

The data that support the findings of this study will be available from the corresponding author upon reasonable request in the form of statistical package for social sciences (SPSS).

## Abbreviations

BLH: Black Lion Hospital
CDC: Centers for Disease Control
EHR: Electronic Health Record
EMR: Electronic Medical Record
FMoH: Federal Ministry of Health
IT: Information Technology
ICT: Information and Communication Technology
OPD: Out Patient Department
UPS: Uninterruptable Power Supply

## References

[1] Ross DS, Venkatesh R (2016). Role of hospital information systems in improving healthcare quality in hospitals. Indian J Sci Technol.

[2] Alotaibi YK, Federico F (2017). The impact of health information technology on patient safety. Saudi Med J.

[3] Luna D, Almerares A, Mayan JC, Gonzalez-Bernaldo-de-Quiros F, Otero C (2014). Health informatics in developing countries: going beyond pilot practices to sustainable implementations: a review of the current challenges. Healthc Inform Res.

[4] WHO (2012). Management of patient information: trends and challenges in Member States: based on the findings of the second global survey on eHealth. Global Observatory for eHealth Series, v. 6.

[5] Rihab H, Kirsten V, Michele C (2014). Progress and challenges in the implementation of electronic medical records in Saudi Arabia: a systematic review. Health Inform Int J.

[6] Shahmoradi L, Darrudi A, Arji G, Nejad AF (2017). Electronic health record implementation: a SWOT analysis. Acta Med Iran.

[7] Ahmadi H, Nilashi M, Almaee A, Soltani M, Zare M, Sangar AB (2016). Multi-level model for the adoption of hospital information system: a case on Malaysia. J Soft Comput Decis Support Syst.

[8] Ohuabunwa EC, Sun J, Jubanyik KJ, Wallis LA (2016). Electronic medical records in low to middle income countries: the case of Khayelitsha Hospital, South Africa. Afr J Emerg Med.

[9] Mohamadali Noor A, Zahari NA (2017). The organization factors as barrier for sustainable health information systems (HIS): a review. 4th information systems international conference 2017, ISICO 2017, 6–8 November 2017, Bali, Indonesia. Procedia Comput Sci.

[10] Msiska KEM, Kumitawal A, Kumwenda B (2017). Factors affecting the utilization of electronic medical records system in Malawian central hospitals. Malawi Med J.

[11] Raut A, Yarbrough C, Singh V, Gauchan B, Citrin D, Verma V (2018). Design and implementation of an affordable, public sector electronic medical record in rural Nepal. J Innov Health Inform.

[12] Joukes E, de Keizer NF, de Bruijne MC, Abu-Hanna A, Cornet R (2019). Impact of electronic versus paper-based recording before EHR implementation on health care professionals’ perceptions of EHR use, data quality, and data reuse. Appl Clin Inform.

[13] King J, Patel V, Jamoom EW, Furukawa MF (2014). Clinical benefits of electronic health record use: national findings, part II. Health Serv Res.

[14] Joukes E, Cornet R, Abu-Hanna A, Bruijne-De M, Keizer-De N, Cornet R, Stoicu-Tiradar L, Horbst A (2015). End-user expectations during an electronic health record implementation: a case study in two academic hospitals. Digital healthcare empowering europeans. European Federation for Medical Informatics (EFMI).

[15] Jawhari B, Ludwick D, Keenan L, Zakus D, Hayward R (2016). Benefits and challenges of EMR implementations in low resource settings: a state-of-the-art review. BMC Med Inform Decis Mak.

[16] Gyamfi A, Mensah Kofi A, Oduro G (2017). Barriers and facilitators to electronic medical records usage in the emergency centre at Komfo Anokye Teaching Hospital, Kumasi-Ghana. Afr J Emerg Med.

[17] Khalifa M (2013). Barriers to health information systems and electronic medical records implementation: a field study of saudi arabian hospitals. Procedia Comput Sci.

[18] Jaillah MG, Almarie B, Kristelle SM, Adrian G (2017). Barriers to electronic health record system implementation and information systems resources: a structured review. Procedia Comput Sci.

[19] Peace U, Kato N, Assouman N, Anne K, Moses I, Julienne M (2017). Health care consumer’s perception of the electronic medical record (EMR) system within a referral hospital in Kigali, Rwanda. Rwanda J Ser F Med Health Sci.

[20] Fritz F, Tilahun B, Dugas M (2015). Success criteria for electronic medical record implementations in low-resource settings: a systematic review. J Am Med Inform Assoc.

[21] Pantaleoni JL, Stevens LA, Mailes ES, Goad BA, Longhurst CA (2015). Successful physician training program for large scale EMR implementation. Appl Clin Inf.

[22] Muinga N, Magare S, Monda J, Kamau O, Houston S, Fraser H (2018). Implementing an open source electronic health record system in Kenyan health care facilities: case study. JMIR Med Inform.

[23] Tierney WM, Sidle JE, Diero LO (2015). Assessing the impact of a primary care electronic medical record system in three Kenyan rural health centers. J Am Med Inform Assoc.

[24] Gagnon MP, Simonyan D, Ghandour EK, Godin G, Labrecque M, Ouimet M (2016). Factors influencing electronic health record adoption by physicians: a multilevel analysis. Int J Inform Manag.

[25] Ologeanu-Taddei R, Morquin D, Vitari C (2016). Perceptions of an electronic medical record (EMR): lessons from a French longitudinal survey. Procedia Comput Sci.

[26] Sidek YH, Martins JT (2017). Perceived critical success factors of electronic health record system implementation in a dental clinic context: an organizational management perspective. Int J Med Inform.

[27] Schopfi TR, Nedrebo B, Hufthammer KO, Daphu IK, Laerum H (2019). How well is the electronic health record supporting the clinical tasks of hospital physicians? A survey of physicians at three Norwegian hospitals. BMC Health Serv Res.

[28] Tiwari P, Saxena VP, Mishra RG, Bhavsar D (2015). Wireless sensor networks: introduction, advantages, applications and research challenges. HCTL Open Int J Technol Innov Res.

