# Supplementary material for: Implementation challenges and perception of care providers on Electronic Medical Records at St. Paul’s and Ayder Hospitals, Ethiopia
Source: BMC Med Inform Decis Mak. 2021 Nov 2;21:306. doi: 10.1186/s12911-021-01670-z (PMC8561912; doi:10.1186/s12911-021-01670-z)
Supplement: Supplementary file 1 — Additional file 1 Title of data: Questionnaire and Interview guide for implementation challenges and perception of care providers on EMR at St. Paul’s and Ayder Hospitals, Ethiopia. Description of Data: A structured and pre-validated questionnaire which contains sections on Socio-Demographics; Knowledge, Perceived Benefits and Usefulness; and Perception and Willingness; whereas a pre-distributed interview guide employed to interview the key informants assuming to get more detailed information in support of findings from the questionnaire [file 12911_2021_1670_MOESM1_ESM.docx]

**Additional File 1:**

**Questionnaire and Interview Guide for implementation challenges and perception of care providers on EMR at St. Paul’s and Ayder Hospitals, Ethiopia.**

**Questionnaire**

**Part A. Demographic Profile**

1. Respondents Institution/hospital _____________________

2. Professional Category

a. GP b. Specialist c. Nurse d. Lab Technician

e. Pharmacist

3. Age Range a. 21 - 30 b. 31 – 40 c. 41 – 50 d. 51 – 60

4. Sex a. Male b. Female

5. Service year in the institution a. 1-5 b. 6-10 c. 11-15 d. >15

**Part B. Knowledge, Perceived Benefits, Availability & Usefulness of EMR systems**

1. How much do you know about electronic medical record (EMR) systems?

a. Almost nothing b. Somewhat c. A few things d. A great deal

2. What are your expectations of the EMR system?

a. The EMR system will improve productivity and office efficiency starting on day one.

b. The EMR system will improve productivity and office efficiency over time as the practice becomes accustomed to using it.

c. The EMR system is just a replacement for traditional paper-based patient folders.

d. The EMR system is unlikely to improve productivity and office efficiency.

3. Please rate your computer skills.

a. Poor b. Medium c. Good d. Excellent

4. Do you have access to Internet in your working area? a. Yes b. No

5. For what purpose do you use the Internet?

a. E-Mail b. Patient care related information c. Drug information d. Other business

6. Adopting and learning a new computer technology requires patience and a commitment to training to use all features effectively. How many hours of training are you willing to devote to learning the EMR system? a. 4 b. 8 c. 12 d. 16 e. 20

7. Have you got training on EMR in the past?

a. Yes b. No

8. What should be the staff’s role in ensuring successful EMR implementation?

a. The practicing physicians have a major role in successful implementation.

b. The nurses and Physicians will drive usage and successful implementation.

c. All staff should work cooperatively for success

9. Do you electronically share medical information about your patients with other health care entities or colleagues? A. Yes b. No

If you answered Yes to Question 9 – Do you share information using (circle appropriate responses): a. EHR/EMR b. E-mail c. Fax d. Phone

10. Do you have EMR installed computer in your working area? a. Yes b. No

11. Do you believe EMR system will improve communication among health care providers?

a. Yes b. No c. Not Applicable

12. Do you think the EMR system is complete to capture all patient encounters at the hospital outpatient clinic? a. Yes b. No c. Not Applicable

13. The extent to which EMR will change the process of patient care?

a. Significantly b. Small degree c. Not at all

14. The extent to which EMR will change the quality of care?

a. Will Improve b. Decrease c. No change

15. EMR has a benefit of increasing practice productivity (Patients per day). a. Yes b. No

16. EMR has a benefit of decreasing the work load and enhancing efficiency of providers.

a. Yes b. No

17. What do you think of barriers of EMR implementation process? (Choose all appropriate).

a. Low user’s acceptance b. Poor Project management c. Poor ICT infrastructures d. lack of training and follow up e. Poor ICT infrastructures and lack of training and follow up f. poor project management, poor ICT infrastructure & lack of training & follow up g. Low user acceptance, poor ICT infrastructure & lack of training & follow up h. Low user acceptance, poor project management, poor ICT infrastructure & lack of training & follow up

18. What do you think of success factors of EMR implementation? (Choose all appropriate).

a. Commitment and involvement of all stakeholders b. Clear long term perspective, endurance and patience c. Commitment and involvement of medical staff d. Good organizational change management, interdisciplinary team with IT experience & training, and clear incentives e. Commitment at the highest level of management f. commitment and involvement of medical staff and highest level management g. Commitment of stakeholders, medical staff, highest management, long term perspective & change management

19. Why the EMR system implementation failed to continue at your Hospital? (If the question applicable to you, circle all you think are appropriate)

a. Lack of appropriate training and follow up b. Lack of management commitment

c. Poor network infrastructure d. Hardware/software problem

e. The EMR system was very complex f. Lack of interest among care providers

g. Lack of champion for the system h. The EMR system does not fully address my needs to perform my day to day tasks i. Not Failed in my institution j. Lack of training & management commitment, poor network connectivity & hardware/software issue k. Poor network, lack of interest, among care providers & lack of Champion for the system

20. If you are using EMR system how do you evaluate patient–providers’ communication?

a. Improved b. Decreased

21. Barrier to implementation of EMR in my hospital is Lack of awareness of the importance and benefits of using EMRs? a. Yes b. No

22. Are you able to input data to the EMR system? a. Yes b. No

23. Do you think service turnaround time (time from test request, sample collection, report generation and receipt of report by clinician) would be improved/decreased by using EMR system?

a. Yes b. No

24. If your answer for question 23 is Yes, indicate by how much the time will be improved for the patient? a. By 30-minute b. By 1-hour c. By 2 hours d. More than 2 hours

25. Do you think system down time (periods of time during which an EMR system, server or network is shut off or unavailable for use) affect your clinical practice?

a. very minimum effect b. high effect c. serious effect d. no effect at all

26. How do you rate the EMR system integration with PACS & Laboratory system interface?

a. Fully integrated b. Partially integrated c. Not integrated

27. How do you think the EMR system effect on patients waiting time?

a. Expect reduction of patient waiting time b. Increased patient waiting time c. No change

28. EMR system is safe and secured as compared to a paper based medical record system.

a. Yes b. No

29. What was your level of satisfaction in using the EMR system at your hospital?

a. Very satisfied b. Satisfied c. Dissatisfied d. Very dissatisfied

e. EMR system installed but not used/implemented

30. For what purpose do you use the EMR among the following tasks? (Chose all appropriate)

a. Review patient history b. Follow the result of particular test c. Obtain the result from new test d. Obtain information on investigation e. Order clinical lab/x-ray/ CT/Ultrasound investigation and obtain the result f. Not applicable

**Part C.** **Perception towards an EMR System and Willingness to use** (Select the number that represents how you feel about EMR)

| **No.** | **Item** | **Strongly Disagree** | **Disagree** | **Don’t Know** | **Agree** | **Strongly Agree** |
| --- | --- | --- | --- | --- | --- | --- |
| 1 | It would be easy for me to become skillful at using EMRs | 1 | 2 | 3 | 4 | 5 |
| 2 | I prefer EMR over paper charting | 1 | 2 | 3 | 4 | 5 |
| 3 | Patient information obtained from EMR system is more complete as compared to Paper medical record | 1 | 2 | 3 | 4 | 5 |
| 4 | My involvement at the system design and during the EMR implementation phase will make the EMR more useful to me | 1 | 2 | 3 | 4 | 5 |
| 5 | I would like to use EMRs in my work in future | 1 | 2 | 3 | 4 | 5 |

**Thank You!**

**Interview Guides**

**Interview Guide for EMR system representatives (SPHMMC/ Ayder Hospital)**

1.When was the starting date of the project? _________

2. Number of trained staff to manage the system_________

3. How is the ICT infrastructure in general?

4. What is the current status of the system?

5. Do you think the system is cost-effective?

6. In which area does the EMR system mostly used in your hospital?

7. How is the awareness level of health care providers to the system?

8. Is there any resistance to the system from care providers/any user?

9. What problems do you encountered in implementing the EMR system?

10. what do you think are factors for failure & success?

11. Is the system user friendly?

12. Do you think the system is complete for the required patient encounter?

13. What do you think of service turnaround time?

14. How do you explain system downtime effects on patients as well as providers?

15. Have you ever come through any patient/user complaints on the system?

16. Any suggestion? ____________________________

**Interview Guide for Hospital Provosts & Medical Directors**

1. What was your objective in implementing this system? Is the objective achieved so far?

2. What do you think of staff awareness and knowledge on the system?

3. Staff attitude and perception towards the system?

4. Do you think you have dependable ICT infrastructure? If no, do you Expect improvement in a near future?

5. Do you think the system is cost-effective?

6. Do you think the system is easy to use/user friendly?

8. Have you ever encountered patient/user complaints on the system?

9. What are the reasons for your success/ failure?

10. Do you have enough trained staff to run the system?

11. Any suggestion including best alternative system for EMR?

**Interview Guide for IT support staffs**

1.What type of problems you encountered on the system?

2. What type of support you provided / received so far on the system?

3. Do you think the software system is easily adoptable/ manageable?

4. Have you faced hardware problems/failures including the server?

5. Do you know any best alternative system than Smart Care/Tena care?

6. How do you rate the ICT infrastructure set up in your hospital?

7. Any suggestion for system improvement?

**Thankyou**
